# TP53 mutated AML subclones exhibit engraftment in a humanized bone marrow ossicle mouse model

**DOI:** 10.1007/s00277-020-03920-y

**Published:** 2020-01-30

**Authors:** Gabriel Pabst, Karin Lind, Ricarda Graf, Armin Zebisch, Friedrich Stölzel, Konstanze Döhner, Ellen Heitzer, Andreas Reinisch, Heinz Sill

**Affiliations:** 1 Division of Hematology, Department of Internal Medicine, Medical University of Graz, Auenbruggerplatz 38, 8036 Graz, Austria; 2 Institute of Human Genetics, Medical University of Graz, Graz, Austria; 3 Otto Loewi Research Center for Vascular Biology, Immunology and Inflammation, Division of Pharmacology, Medical University of Graz, Graz, Austria; 4 Department of Internal Medicine I, University Hospital Carl Gustav Carus, Dresden University of Technology Dresden, Dresden, Germany; 5 Department of Internal Medicine III, University Hospital of Ulm, Ulm, Germany

Dear Editor,

Despite extensive efforts to develop novel therapies, the prognosis of patients with acute myeloid leukemia (AML) is still poor. One of the reasons is the genetic heterogeneity of AML with the majority of patients exhibiting distinct mutational subclones and diverse biological characteristics [1, 2]. *TP53* mutated AMLs are frequently resistant to intensive treatments and we recently showed that subclonal *TP53* mutations confer an equally devastating prognosis [3]. This observation lead us to hypothesize that *TP53* mutated subclones display characteristics of leukemic stem cells (LSCs) thus contributing to relapse or resistant disease. We, therefore, tested LSC properties of these subclones in a recently developed, highly sensitive humanized bone marrow (BM) ossicle xenotransplantation mouse model [4, 5].

Patient specimens, experimental methods, and ethical approvals are described in detail in the Supplementary Data. Briefly, BM-derived mesenchymal stromal cells (MSCs) from healthy donors were expanded in vitro and subcutaneously injected into four sites of immunodeficient NOD/SCID/γ^null^ (NSG) mice. These MSCs underwent endochondral ossification leading to the formation of a humanized BM ossicle microenvironment. We transplanted three diagnostic, T cell depleted AML specimens into ossicle-bearing NSG mice either by tail vein or direct intraossicle injection. Two specimens showed subclonal *TP53* mutations with a variant allele frequency (VAF) of <20%, one a clonal *TP53* mutation serving as control (Supplementary Table 1). Mice were sacrificed 16–18 weeks post transplantation and humanized ossicles as well as mouse BM were analyzed for the engraftment of human leukemia. Leukemia engraftment was analyzed by multicolor flow cytometry, and different engrafted cell populations were sorted based on the expression of hCD45, hCD33 and hCD19 (Supplementary Fig. 1). gDNA of sorted cells was analyzed for patient-specific *TP53* and cooperating mutations using high-resolution mutation profiling allowing for the detection of mutations with a VAF of <0.1% [6].

All three AML specimens showed engraftment in humanized ossicles as well as mouse BM. However, human cells preferentially engrafted in the humanized ossicle microenvironment (Fig. 1a, Supplementary Fig. 2). For both AML samples with subclonal *TP53* mutations, CD33+ leukemic cells constituted the predominant cell population within the graft. Interestingly, differentiation into CD19+ B-lymphoid cells was also observed and the subclonal *TP53* mutations could be detected in both, the engrafted myeloid and lymphoid compartments (Figs. 1b, c; Supplementary Figs. 3,4; Supplementary Tables 2,3). These data indicate that subclones with *TP53* mutations reveal characteristics of LSCs and pre-leukemic hematopoietic stem cells (pHSCs) being in line with our previous report on clonal *TP53* mutations in AML [7]. Stem cell features may contribute to the fact that AML patients with subclonal *TP53* mutations do also face an adverse prognosis since LSCs and pHSCs are considered less vulnerable to cytotoxic therapy ultimately giving rise to relapsed or resistant disease [8]. Similarly, expansion of clones with *TP53* aberrations was also shown in murine models exposed to genotoxic stress [9, 10]. Finally, our data re-emphasize the usefulness of the humanized BM ossicle mouse model, particularly for the engraftment of small myeloid clones.

**Fig. 1:**
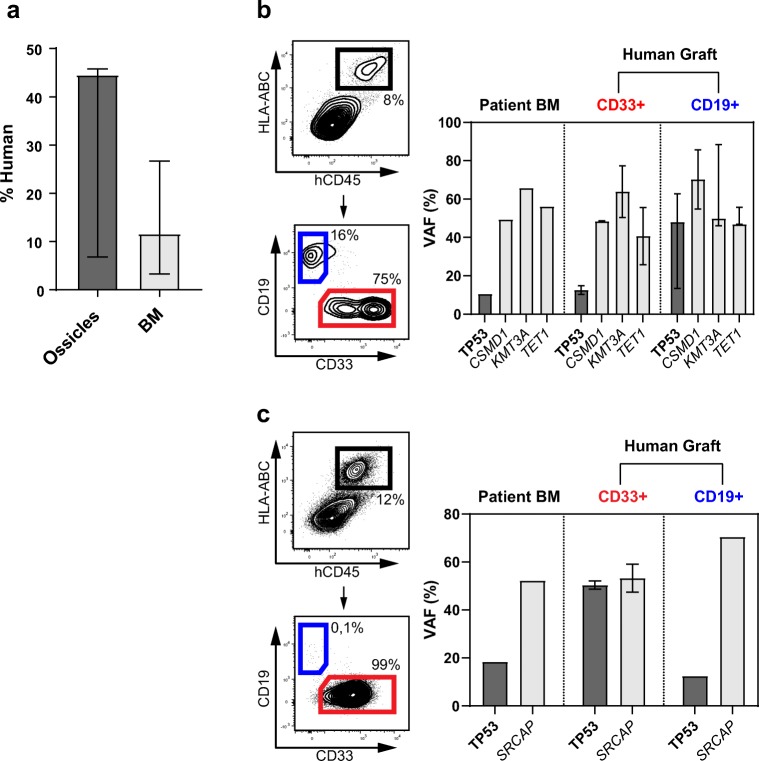
Engraftment of *TP53* mutated AML specimens in a humanized bone marrow ossicle mouse model. a, Median human engraftment of AML specimens following tail vein injection in humanized bone marrow ossicles and mouse bone marrow of three mice 16 weeks post transplantation. b,c, Engraftment of two AML specimens with subclonal *TP53* mutations. The left panels depict human hematopoietic cells being hCD45+ and HLA-ABC+ and sorted into CD33+ myeloid and CD19+ B-lymphoid cells. The right panels depict variant allele frequencies (VAFs) of the particular *TP53* and cooperating mutations in the respective compartments. Error bars denote 95% confidence intervals. Abbreviation: BM, bone marrow

## Electronic supplementary material

ESM 1 (PDF 2815 kb)
