# Supplementary material for: TP53 mutated AML subclones exhibit engraftment in a humanized bone marrow ossicle mouse model
Source: Ann Hematol. 2020 Jan 30;99(3):653–5. doi: 10.1007/s00277-020-03920-y (PMC7060155; doi:10.1007/s00277-020-03920-y)
Supplement: Supplementary file 1 — (PDF 2815 kb) [file 277_2020_3920_MOESM1_ESM.pdf]

Supplementary Data

TP53 mutated subclones in AML exhibit engraftment in a humanized bone marrow ossicle mouse model

Gabriel Pabst,[1] Karin Lind,[1] Ricarda Graf,[2] Armin Zebisch,[1,3] Friedrich Stölzel,[4] Konstanze Döhner,[5] Ellen Heitzer,[2] Andreas Reinisch[1] and Heinz Sill[1]

[1]Division of Hematology, Medical University of Graz, Graz, Austria
[2]Institute of Human Genetics, Medical University of Graz, Graz, Austria
[3]Otto Loewi Research Center for Vascular Biology, Immunology and Inflammation, Division of Pharmacology, Medical University of Graz, Graz, Austria
[4]Department of Internal Medicine I, University Hospital Carl Gustav Carus, Dresden University of Technology Dresden, Dresden, Germany.
[5]Department of Internal Medicine III, University Hospital of Ulm, Ulm, Germany

Patients and Methods

Primary leukemia specimens

The study was approved by the ethics committee of the Medical University of Graz, Austria, and the University of Ulm, Germany, and written informed consent was obtained from all patients. All specimens were collected at AML diagnosis and processed by Ficoll density gradient centrifugation to enrich for mononuclear cells (MNCs). MNCs were analyzed by a targeted sequencing approach focusing on genes associated with myeloid neoplasms as described previously [1,2]. Variant allele frequencies (VAFs) were determined by counting the number of variant reads divided by the number of total reads.

Sample	Source	Blasts (%)	Gene	AA change	VAF (%)
**5273**	PB	39	*TP53*	p.P152L	52,1
			TP53	p.M237I	45,8
**9254**	BM	30	*TP53*	p.C176F	10.43
			CSDM1	p.T144S	49.34
			KMT2A	p.I1870T	65.81
			TET1	p.G1802R	56
**9260**	BM	36	*TP53*	p.R273H	18,37
			SRCAP	p.V890I	52,15

Supplementary Table 1. Characteristics of the AML specimens used for xenotransplantation. Abbreviations: AA, amino acid; VAF, variant allele frequency; PB, peripheral blood; BM, bone marrow.

Humanized BM ossicle niche formation in NSG mice

All animal experiments were conducted in accordance with a protocol of the Institutional Animal Care and Use Committee at Medical University of Graz, Austria and approved by the Austrian Ministry for Science (GZ: BMWFW-66.010/0018-V/3b/2018). FELASA guidelines to minimize animal distress and suffering were strictly followed [3]. Humanized BM ossicles were generated as previously described [4,5]. BM-derived mesenchymal stromal cells (MSCs) from healthy donors were expanded *in vitro*, harvested, re-suspended in 60 µl of pooled human platelet lysate (pHPL) and admixed with 240 µl of extracellular matrix (Angiogenesis assay kit; Millipore). This mixture was then subcutaneously injected into the flanks - four sites - of immunodeficient NOD/SCID/γ^{null} (NSG) mice (Jackson Laboratory). Following MSC application, mice received daily subcutaneous injections of human parathyroid hormone (PTH (1–34); R&D Systems; 40 µg/kg body weight; dorsal neck fold) for 28 consecutive days. Eight to 12 weeks after MSC application, mice were evaluated for the development of humanized BM ossicles by inspection and palpation.

Xenotransplantation

Conditioning of ossicle-bearing NSG mice was performed by irradiation with 130 rad 12-24 hours before transplantation. Human AML specimens were thawed in RPMI supplemented with 10% FBS and DNase (20U/ml; Sigma) and T-cell depleted on cell separation magnet using anti-human CD3 magnetic particles (both Becton Dickinson [BD]). A total of 0,43x10^6 to 1,0x10^6 cells were transplanted by direct intraossicle injection of one ossicle per mouse (cells re-suspended in 20 µl RPMI) or by tail vein injection (cells re-suspended in 100 µl RPMI).

Assessment of human engraftment and sorting of human engrafted cell compartments

Human engraftment was assessed as previously defined [6]. Short-term engraftment was assessed 8-10 weeks after transplantation in BM by aspiration via femur and the transplanted ossicle. All samples were lysed (red blood cell lysis buffer, ebioscience) to remove contaminating RBCs. Thereafter, cells were blocked for nonspecific-antibody binding (5 minutes at room temperature) using the FCR blocking reagent (Biolegend) and stained (30 minutes at 4 °C in the dark) with fluorochrome-conjugated monoclonal antibodies: CD45-KromeOrange (Dilution 1:80; Beckman Coulter; clone J33), CD33-FITC (Dilution 1:40; Beckman Coulter; clone D3HL60.251), CD19-PC7 (Dilution 1:25; Beckman Coulter; Clone J3-119), mTer-119-PerCP-Cy5.5 (Dilution 1:25; BD; Clone Ter-119), mCD45-PE (Dilution 1:200; Pharmingen; Clone 30-F11), CD3-APC-Cy7 (Dilution 1:160; BD; clone Sk7), HLA-ABC-Pacific Blue (Dilution 1:160; Biolegend; clone Wg/32) and CD34-APC (Dilution 1:10; Beckman Coulter; clone 581). Multicolor flow cytometry was performed using a BD FACSAria III and post hoc analyses of flow cytometric data were done using FlowJo 10.5.3. (BD).

Long-term engraftment was assessed 16-18 weeks after transplantation in BM, transplanted ossicles and non-transplanted ossicles. Blood was collected by cardiac punctures immediately after euthanasia. To isolate cells, excised ossicles were crushed using a mortar and pestle, and mouse BM (from both femora, tibiae and humeri) was flushed using RPMI. Blocking and staining was done as described in the short-term engraftment analysis using the same antibody panel. Different cell populations were sorted based on their expression of hCD45, CD33 and CD19 on a BD FACSAria III using the sorting strategy shown in Supplementary Fig. 1. gDNA of sorted cell populations was extracted using the QIAamp DNA Micro Kit (Qiagen).

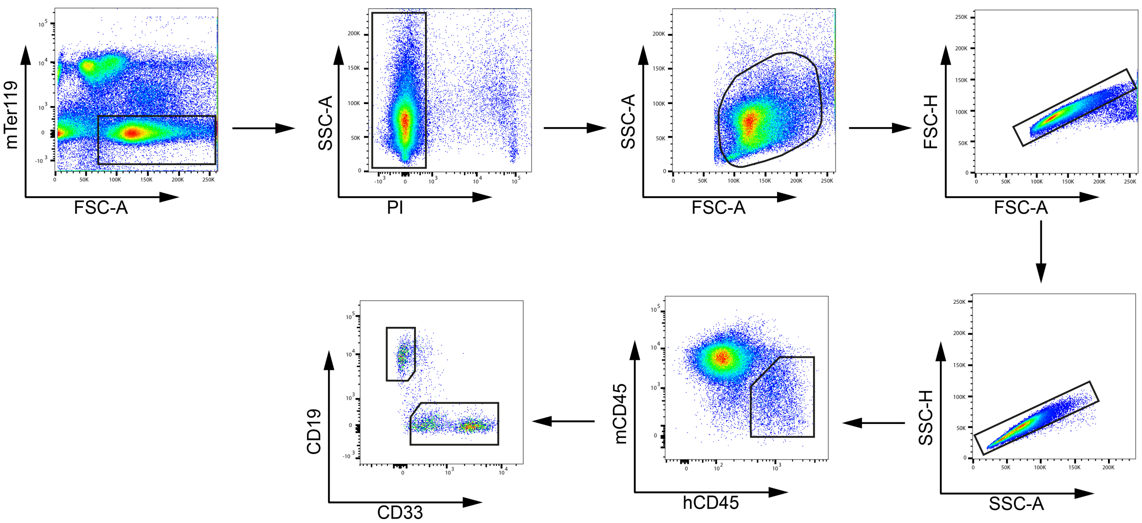

Supplementary Fig. 1. Strategy used to sort engrafted human myeloid and lymphoid cells. Non-mouse erythroid cells are identified by their lack of mTer119 expression, live cells are discriminated based on their propidium iodide (PI) binding, single cells are discriminated from doublets using SSC-H/SSC-A and FSC-H/FSC-A plots. Human hematopoietic cells are mCD45- and hCD45+, myeloid cells are CD33+ and B-lymphocytes are CD19+. Plots of one representative xenograft sample are depicted.

High resolution mutation profiling

Patient-specific *TP53* and cooperating mutations were analyzed in xenograft samples using the recently published SiMSen-seq method. This method is based on molecular barcoding of individual DNA template strands to track all sequencing reads back to a single original template and correct for PCR errors during library preparation which allows a detection of mutations with variant allele frequencies of <0.1% [7]. For each mutation, three target primer pairs were designed considering product size, primer size, annealing temperature and complementarity. All primers were tested with a high-quality control DNA (Promega) using quantitative realtime-PCR (qRT-PCR) including melt curve analysis by comparing the performance of the target primers to a control assay (TP1). Assays with cycle of quantification (Cq) values closest to the control assay were further evaluated after the addition of the barcoded hairpin primer sequences. Finally, all assays that passed the quality criteria were multiplexed with a final concentration of 1µM each and re-evaluated in a low depth sequencing run to check for a balanced coverage. A minimum of 10 ng is recommended as input for the SiMSen-seq. In the cases were input DNA amount was below 10ng, 2.5µl of DNA were whole genome amplified using the illustra GenomiPhi V2 DNA Amplification Kit (GE Healthcare) following the manufacturer´s instructions and subsequently purified using AMPure Beads XP (Beckman Coulter). Amplified samples were thereafter quantified using Qubit High Sensitivity Kit. SimSen-Seq libraries were then prepared using an

average of 114ng (range, 50-200ng) of input DNA and quality checked on an Agilent Bioanalyzer 7500 Kit. Considering the number of amplicons per assay, libraries were equimolarly pooled together followed by another purification step and quality check. The final concentration of the library pool was determined by qRT-PCR using the QIASeq Library Quant Assay Kit (Qiagen). Sequencing was performed on either Illumina NextSeq or MiSeq using 150bp single-end read mode. Data analysis was performed using the new tool kit Debarcer. Considering only barcode families containing a minimum of 10 reads, an average consensus read depth of 4493 (range, 276-29369) was obtained.

References

1. Elkashef SM, Lin AP, Myers J, Sill H, Jiang D, Dahia PLM, Aguiar RCT. IDH Mutation, Competitive Inhibition of FTO, and RNA Methylation. Cancer Cell. 2017;31(5):619-620.

2. Papaemmanuil E, Gerstung M, Bullinger L, Gaidzik VI, Paschka P, Roberts ND, Potter NE, Heuser M, Thol F, Bolli N, Gundem G, Van Loo P, Martincorena I, Ganly P, Mudie L, McLaren S, O'Meara S, Raine K, Jones DR, Teague JW, Butler AP, Greaves MF, Ganser A, Dohner K, Schlenk RF, Dohner H, Campbell PJ. Genomic Classification and Prognosis in Acute Myeloid Leukemia. N Engl J Med. 2016;374(23):2209-2221.

3. Kilkenny C, Browne WJ, Cuthill IC, Emerson M, Altman DG. Improving bioscience research reporting: the ARRIVE guidelines for reporting animal research. PLoS Biol. 2010;8(6):e1000412.

4. Reinisch A, Thomas D, Corces MR, Zhang X, Gratzinger D, Hong WJ, Schallmoser K, Strunk D, Majeti R. A humanized bone marrow ossicle xenotransplantation model enables improved engraftment of healthy and leukemic human hematopoietic cells. Nat Med. 2016;22(7):812-821.

5. Reinisch A, Hernandez DC, Schallmoser K, Majeti R. Generation and use of a humanized bone-marrow-ossicle niche for hematopoietic xenotransplantation into mice. Nat Protoc. 2017;12(10):2169-2188.

6. Jan M, Snyder TM, Corces-Zimmerman MR, Vyas P, Weissman IL, Quake SR, Majeti R. Clonal evolution of preleukemic hematopoietic stem cells precedes human acute myeloid leukemia. Sci Transl Med. 2012;4(149):149ra118.

7. Stahlberg A, Krzyzanowski PM, Egyud M, Filges S, Stein L, Godfrey TE. Simple multiplexed PCR-based barcoding of DNA for ultrasensitive mutation detection by next-generation sequencing. Nat Protoc. 2017;12(4):664-682.

Results

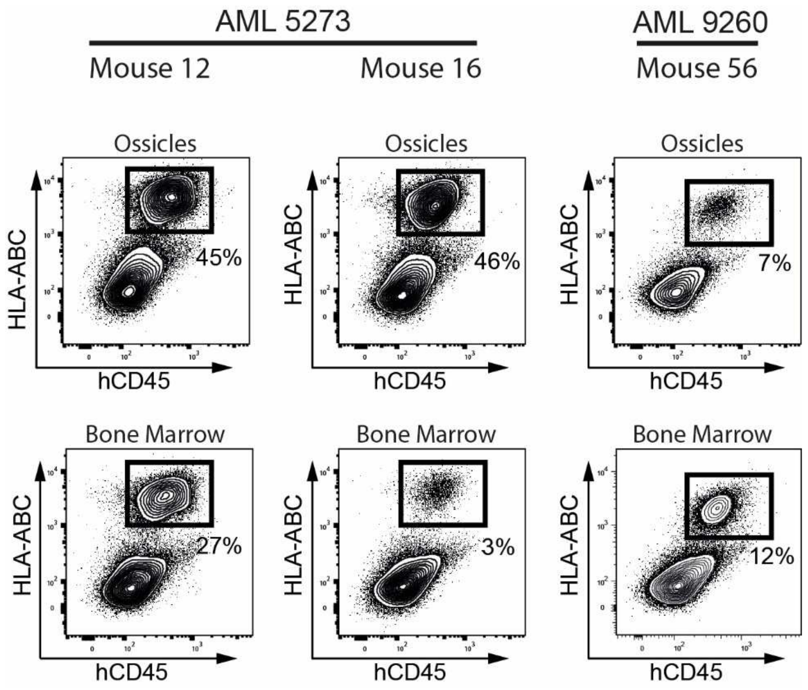

Supplementary Fig. 2. Engraftment analyses of three ossicle-bearing mice transplanted with primary AML patient specimens (AML 5273, AML 9260) by tail vein injections. Gates within the plots show the percentage of human hematopoietic cells (hCD45+, HLA-ABC+). Upper row depicts human engraftment in ossicles, the bottom row in the mouse bone marrow.

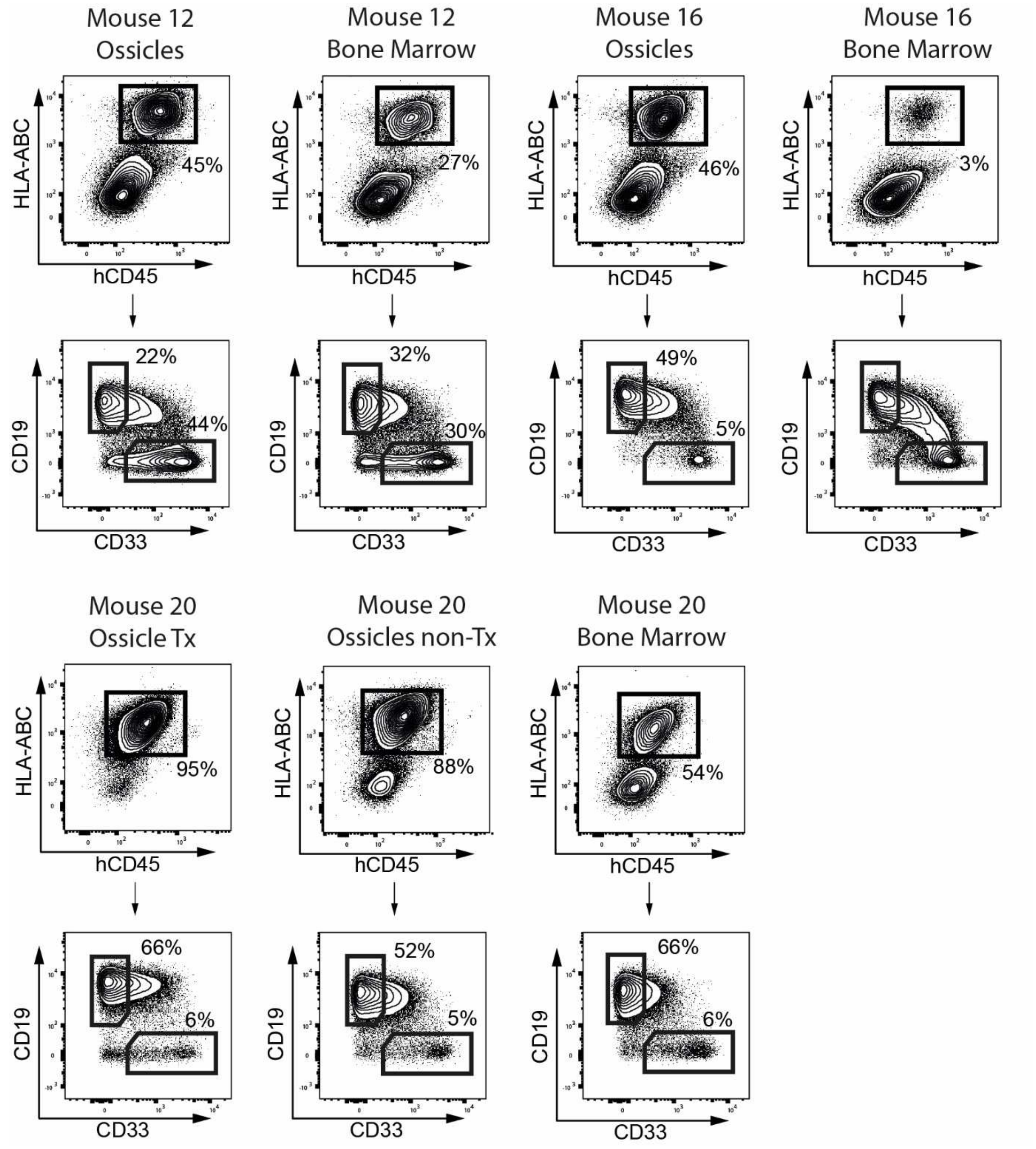

Supplementary Fig. 3. Engraftment analyses of mice transplanted with primary AML patient specimen (AML 5273) carrying two clonal *TP53* mutations. Mouse 12 and 16 were transplanted by tail vein injections, whereas mouse 20 was transplanted by direct intraossicle injection. Gates within the contour plots show the percentages of human hematopoietic cells (hCD45+, HLA-ABC+, upper rows) and percentages of myeloid (CD33+) and B-lymphoid cells (CD19+, lower rows) within the human graft in transplanted ossicles (ossicle Tx), non-transplanted ossicles (ossicle non-Tx) and the bone marrow..

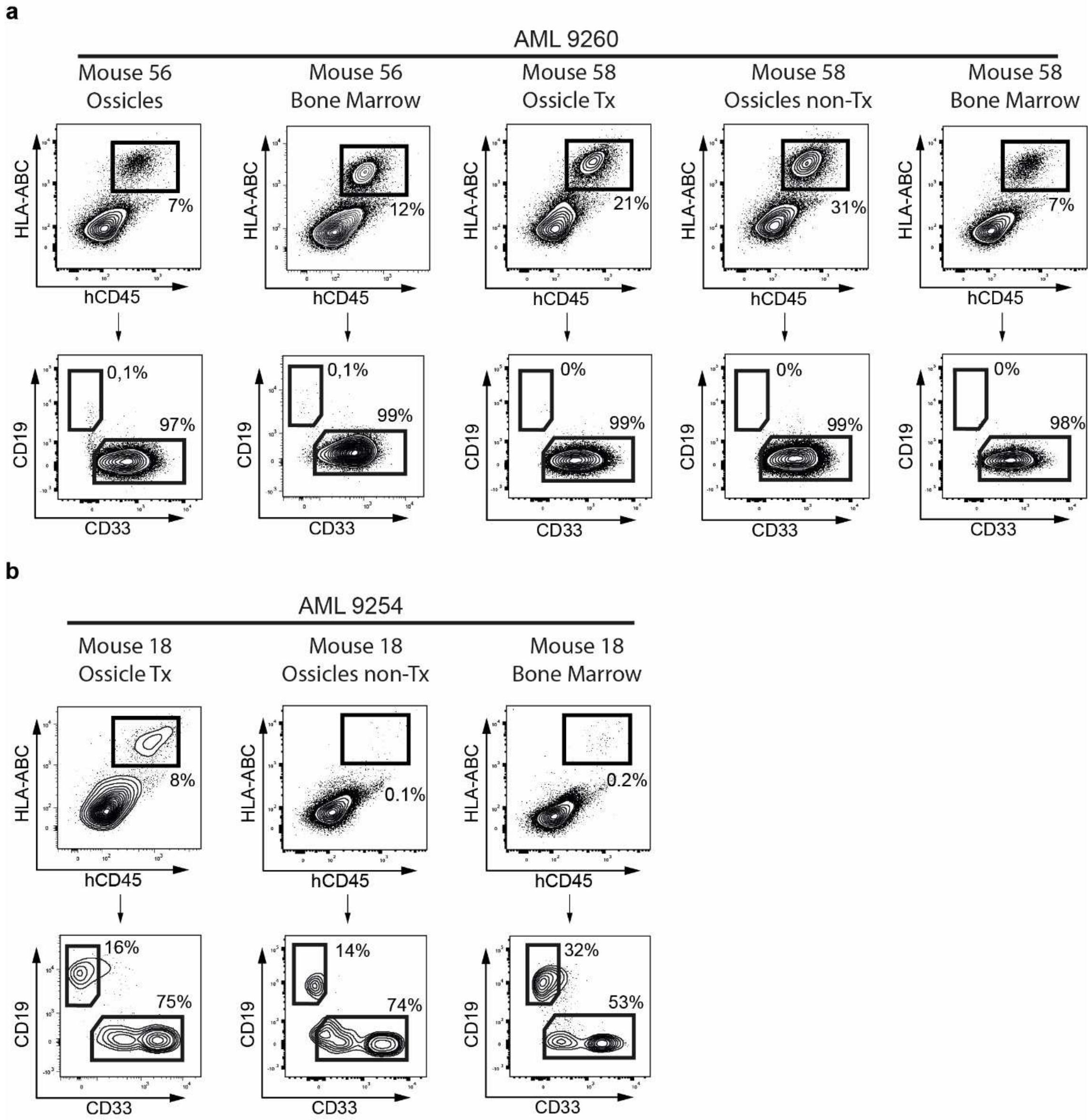

Supplementary Figure 4. Engraftment analyses of ossicle-bearing mice transplanted with primary AML patient specimens (AML 9260, AML 9254) carrying subclonal *TP53* mutations. Mouse 56 was transplanted by tail vein injection whereas mice 58 and 18 were transplanted by direct intraossicle injection. Gates within contour plots show the percentages of human hematopoietic cells (hCD45+, HLA-ABC+, upper rows) and percentages of myeloid (CD33+) and B-lymphoid cells (CD19+, lower rows) of the human graft in mice transplanted with AML sample 9260 **(a)** AML sample 9254 **(b)**.

Mouse	Sample	Mutation	VAF [%]	Depth
18	Tx Ossicle CD33+	TP53 p.C176F	10,32	10383
		CSMD1 p.T144S	48,69	1031
		KMT2A p.I1870T	50,41	5271
		TET1 p.G1802R	55,62	5732
18	Tx Ossicle CD19+	TP53 p.C176F	13,44	20859
		CSMD1 p.T144S	54,82	1306
		KMT2A p.I1870T	49,79	13225
		TET1 p.G1802R	46,91	13444
18	non-Tx Ossicle CD33+	TP53 p.C176F	14,77	1097
		CSMD1 p.T144S	low depth	45
		KMT2A p.I1870T	77,38	2140
		TET1 p.G1802R	25,80	976
18	non-Tx Ossicle CD19+	TP53 p.C176F	62,80	1465
		CSMD1 p.T144S	low depth	179
		KMT2A p.I1870T	88,40	1034
		TET1 p.G1802R	46,86	1626
18	Bone Marrow CD33+	TP53 p.C176F	29,10	730
		CSMD1 p.T144S	48,10	276
		KMT2A p.I1870T	52,60	934
		TET1 p.G1802R	48,30	1020
18	Bone Marrow CD19+	TP53 p.C176F	48,03	1393
		CSMD1 p.T144S	85,70	2546
		KMT2A p.I1870T	46,08	29369
		TET1 p.G1802R	55,78	13848

Supplementary Table 2. Sequencing results of xenografts generated from AML 9254. VAF, variant allele frequency.; ossicle Tx, transplanted ossicle; non-Tx ossicle, non-transplanted ossicle.

Mouse	Sample	Mutation	VAF [%]	Depth
56	non-Tx ossicle CD33+	TP53 p.R273H	51,90	3320
		SRCAP p.V890I	53,29	1689
56	Bone Marrow CD33+	TP53 p.R273H	49,89	3311
		SRCAP p.V890I	47,39	1667
56	Bone Marrow CD19+	TP53 p.R273H	12,4	2581
		SRCAP p.V890I	70,4	851
58	Tx ossicle CD33+	TP53 p.R273H	50,32	5860
		SRCAP p.V890I	53,16	2197
58	non-Tx ossicle CD19+	TP53 p.R273H	48,72	4789
		SRCAP p.V890I	59,05	2557
58	Bone Marrow CD33+	TP53 p.R273H	52,11	3299
		SRCAP p.V890I	47,64	1484

Supplementary Table 3. Sequencing results of xenografts generated from AML 9260. VAF, variant allele frequency. Tx ossicle, transplanted ossicle; non-Tx ossicle, non-transplanted ossicle.
